# Supplementary material for: Cognitive Avoidance Is Associated with Decreased Brain Responsiveness to Threat Distractors under High Perceptual Load
Source: Brain Sci. 2023 Apr 5;13(4):618. doi: 10.3390/brainsci13040618 (PMC10136524; doi:10.3390/brainsci13040618)
Supplement: Supplementary file 1 [file brainsci-13-00618-s001.zip › brainsci-2268910-supplementary.pdf]

Table S1: Correlations between coping styles, trait anxiety, and emotional accuracy effects (hit rate neutral minus hit rate emotion) under low and high perceptual load

	Accuracy effect low load		Accuracy effect high load	
	contempt	fear	contempt	fear
MCI-VIG	-.08	-.04	.02	.03
MCI-CAV	.10	.24	-.18	.13
STAI-trait	-.18	-.10	.09	-.04

*p < .05

Table S2: Perceptual load main effects: Brain regions showing different activation under the high load (vs. low load) condition

	Hemisphere	Peak *T*-value	Peak-level $p_{\text{uncorrected}}$	Cluster size (voxels)	Cluster-level p_{FWE}	Peak MNI *x*	*y*	*z*
Low perceptual load > high perceptual load								
All emotional conditions (contempt, fear, neutral)								
None	-	-	-	-	-	-	-	-
Low perceptual load < high perceptual load								
Contempt								
Inferior occipital extending to calcarine	L	7.31	<.001	740	<.001	-21	-88	-8
Precentral gyrus (BA6)	L	4.52	<.001	111	.04	-30	-13	49
Superior parietal gyrus	L	4.51	<.001	106	.04	-21	-58	49
Fear								
Superior and inferior parietal gyrus, extending to the following regions:	L/R	8.90	<.001	9243	<.001	-27	-52	49
bilateral precuneus and cuneus, superior and middle occipital	L/R	8.00	<.001			-24	-64	37
Cerebellum	L/R	7.18	<.001			9	-67	-20
Bilateral middle frontal gyrus, extending to bilateral precentral gyrus (BA6), right medial frontal and anterior cingulate gyrus	L/R	7.09	<.001			-30	-7	52
Bilateral insula	L/R	4.58	<.001			39	20	7
Bilateral thalamus	L/R	5.27	<.001			15	-7	4

(*table continued*)	Hemisphere	Peak *T*-value	Peak-level $p_{\text{uncorrected}}$	Cluster size (voxels)	Cluster-level p_{FWE}	Peak MNI *x*	*y*	*z*
Low perceptual load < high perceptual load (*continued*)								
Neutral								
Middle occipital gyrus, extending to inferior and superior parietal gyrus (BA7), precuneus	L	5.81	<.001	749	<.001	-30	-79	16
Occipital gyrus, extending to cuneus, calcarine	R	5.75	<.001	208	<.01	21	-85	1
Superior parietal gyrus	R	5.00	<.001	178	<.02	27	-64	58

Neuroanatomical labels, hemisphere, peak voxel t- and p-values, cluster extent, cluster-level FWE-corrected p-values, and coordinates in MNI space are presented. Activation clusters are yielded by one-sample t-tests and are significant at $p = .001$ (uncorrected) and a cluster-level threshold of $p_{FWE}<.05$.
